# Comparative proteome and serum analysis identified FSCN1 as a marker of abiraterone resistance in castration-resistant prostate cancer

**DOI:** 10.1038/s41391-023-00713-y

**Published:** 2023-08-26

**Authors:** Anita Csizmarik, Nikolett Nagy, Dávid Keresztes, Melinda Váradi, Thilo Bracht, Barbara Sitek, Kathrin Witzke, Martin Puhr, Ilona Tornyi, József Lázár, László Takács, Gero Kramer, Sabina Sevcenco, Agnieszka Maj-Hes, Boris Hadaschik, Péter Nyirády, Tibor Szarvas

**Affiliations:** 1 Department of Urology, Semmelweis University, Budapest, Hungary; 2 Department of Molecular Biology, Semmelweis University, Budapest, Hungary; 3 Medizinisches Proteom-Center, Ruhr University Bochum, Bochum, Germany; 4 Department of Anesthesia, Intensive Care Medicine and Pain Therapy, University Hospital Knappschaftskrankenhaus Bochum, Bochum, Germany; 5 Center for Protein Diagnostics, Medical Proteome Analysis, Ruhr-University Bochum, Bochum, Germany; 6 Department of Urology, Medical University of Innsbruck, Innsbruck, Austria; 7 Department of Human Genetics, University of Debrecen, Debrecen, Hungary; 8 Biosystems International Kft, Debrecen, Hungary; 9 Department of Urology, Medical University of Vienna, Vienna, Austria; 10 Department of Urology, University Hospital Essen, University of Duisburg-Essen, Essen, Germany

**Keywords:** Predictive markers, Prostate cancer, Cancer therapy

## Abstract

**Background:**

Abiraterone (Abi) is an androgen receptor signaling inhibitor that significantly improves patients’ life expectancy in metastatic prostate cancer (PCa). Despite its beneficial effects, many patients have baseline or acquired resistance against Abi. The aim of this study was to identify predictive serum biomarkers for Abi treatment.

**Methods:**

We performed a comparative proteome analysis on three Abi sensitive (LNCaPabl, LAPC4, DuCaP) and resistant (LNCaPabl-Abi, LAPC4-Abi, DuCaP-Abi) PCa cell lines using liquid chromatography tandem mass spectrometry (LC-MS/MS) technique. Two bioinformatic selection workflows were applied to select the most promising candidate serum markers. Serum levels of selected proteins were assessed in samples of 100 Abi-treated patients with metastatic castration-resistant disease (mCRPC) using ELISA. Moreover, FSCN1 serum concentrations were measured in samples of 69 Docetaxel (Doc) treated mCRPC patients.

**Results:**

Our proteome analysis identified 68 significantly, at least two-fold upregulated proteins in Abi resistant cells. Using two filtering workflows four proteins (AMACR, KLK2, FSCN1 and CTAG1A) were selected for ELISA analyses. We found high baseline FSCN1 serum levels to be significantly associated with poor survival in Abi-treated mCRPC patients. Moreover, the multivariable analysis revealed that higher ECOG status (>1) and high baseline FSCN1 serum levels (>10.22 ng/ml by ROC cut-off) were independently associated with worse survival in Abi-treated patients (*p* < 0.001 and *p* = 0.021, respectively). In contrast, no association was found between serum FSCN1 concentrations and overall survival in Doc-treated patients.

**Conclusions:**

Our analysis identified baseline FSCN1 serum levels to be independently associated with poor survival of Abi-treated, but not Doc-treated mCRPC patients, suggesting a therapy specific prognostic value for FSCN1.

## Introduction

Prostate cancer (PCa) is one of the most frequently diagnosed solid cancers among men worldwide [1]. Abiraterone is a selective and irreversible inhibitor of the enzyme CYP17A1, which is a crucial factor during androgen biosynthesis [2]. Abi has demonstrated improved overall and progression-free survival in both hormone sensitive and castration-resistant metastatic PCa both in chemotherapy-naïve and post-chemotherapy settings [3–6]. However, many patients show primary resistance or develop secondary resistance against this therapy. Currently further therapies with various mechanisms of action are increasingly becoming available providing reasonable options for metastatic castration-resistant prostate cancer (mCRPC) patients. Therefore, predictive biomarkers such as pathologic BRCA1/2 mutations for PARP inhibitors and PSMA-uptake for PSMA radioligand therapy are needed for improved therapeutic decision-making.

In the last years, several Abi resistance mechanisms have been described, which can be classified into two groups; androgen receptor (AR) signaling related and non-AR-related mechanisms [7]. The most common AR-related mechanism is AR copy number gain, which leads to enhanced AR expression resulting in reduced sensitivity to Abi [8]. A further AR-related resistance mechanism is related to specific activating point mutations or splice variants (such as AR-V7) of the AR [9, 10]. In addition, different non-AR-related mechanisms may contribute to Abi insensitivity, e.g. the alterations of DNA repair genes and neuroendocrine transdifferentiation [11, 12]. Additionally, our group has recently found elevated serum ALCAM levels to be associated with shorter survival of Abi- but not Doc-treated patients [13].

The aim of the present study was to identify therapy predictive biomarkers of Abi resistance of PCa. First, we performed comparative proteome analysis on Abi-sensitive and resistant PCa cell lines. This analysis identified 68 significantly, at least two-fold upregulated proteins in Abi resistant cells. Then, we used two different bioinformatics workflows in order to select the most promising candidates. Serum concentrations of selected proteins were determined in Abi-treated mCRPC patients’ samples by using the ELISA method. As Fascin-1 (FSCN1) serum levels are associated with poor survival in Abi-treated patients, its serum concentrations were assessed also in samples of Doc-treated mCRPC men.

## Materials and methods

### Cell culture and reagents

For in vitro experiments, we used the LNCaPabl, LAPC4, and DuCaP human PCa cell lines and their Abi-resistant sublines (LNCaPabl-Abi, LAPC4-Abi, and DuCaP-Abi). Abi-resistant cells were generated by treatment with increasing concentrations of Abi, as described by Puhr et al. [14]. LAPC4 and DuCaP were grown in RPMI 1640 medium supplemented with 10% fetal bovine serum (Biowest, Nuaillé, France), 1% Glutamax (Thermo Fisher Scientific, Darmstadt, Germany) and 1% penicillin/streptomycin (Lonza, Basel, Switzerland). For the LAPC4 cell culture, 1 nM dihydrotestosterone was applied. LNCaPabl was cultured in RPMI 1640 medium supplemented with 10% charcoal stripped FBS (HyClone, GE Healthcare), 1% Glutamax (Thermo Fisher Scientific), and 1% penicillin/streptomycin (Lonza, Basel, Switzerland). Cells were maintained at 37 °C in a humidified atmosphere of 5% CO_2_. The identity of all cell lines was confirmed by short tandem repeat analysis. All experiments were performed with mycoplasma-free cells. Abi (MedChemExpress) was dissolved in EtOH as a 100 mM stock solution and stored at −80 °C.

### Liquid chromatography tandem mass spectrometry (LC-MS/MS) analysis

In order to identify differentially expressed proteins between Abi-sensitive and resistant cell lines, proteome analyses were done using the LC-MS/MS technique. Six technical replicates for each cell line were used for the analysis. Details on LC-MS/MS and protein identification are described in Supplementary Materials and Methods.

### Biomarker selection

In Abi-resistant cells, proteins quantified with minimum two unique peptides and those passing the applied significance thresholds (FDR-corrected *p*-value ≤ 0.05, fold change ≥2) were considered. Two different bioinformatic workflows were used in order to identify the most promising proteins.

First, we applied a workflow, which used the existing prediction programs (SignalP 4.1, SecretomeP 2.0, TargetP 1.1, TMHMM 2.0) and databases (Uniprot, Human Protein Atlas, NCBI, ExoCarta) for the prediction of potentially secreted proteins.

Second, we applied a selection method by scoring the proteins based on their known oncological role and their molecular interactions (number of edges) according to the STRING database. STRING database was used as follows: we conducted a multiple protein search with those proteins that were significantly, at least two-fold upregulated in Abi resistant compared to parental sensitive PCa cells. We investigated which of these proteins have the highest number of interactions with each other. For this, we considered the “known interactions” based on the STRING, which included the 1) interactions between proteins from curated databases and 2) the experimentally determined interactions. In addition, text-mining edges were also considered, if the co-mention in the reference articles raised the functional or physical relationship of proteins. Based on these, we scored our protein list, with 1 as the lowest, and 3 being the highest link numbers.

In addition, we considered the availability of ELISA assays for later serum analyses.

### Patient cohort and sample

Serum samples were collected within one day before Abi treatment between 11/2008 and 05/2015 from 100 mCRPC patients. In addition, serum samples at 3 months after therapy start were also available for 40 Abi-treated patients. Serum samples were collected at the Department of Urology at the Medical University of Vienna and at the Semmelweis University, Budapest. As one of the selected proteins was associated with survival in Abi-treated patients, its serum levels were determined also in serum samples of 69 mCRPC patients who received Doc chemotherapy between 1/2013 and 04/2019. The study was performed in accordance with the ethical standards of the Helsinki Declaration and was approved by the ethical boards of the hospitals (TUKEB 55/2014, ECS 1986/2017). PSA response was defined, according to the Prostate Cancer Clinical Trials Working Group Criteria (PCWG) II, as at least 50% PSA decline from baseline during therapy [15].

### Serum ELISA analyses

Serum concentrations of FSCN1, KLK2 (Kallikrein-2), AMACR (alpha-methylacyl-CoA racemase), and CTAG1A (cancer testis antigen 1A) were measured in 100 Abi-treated patients by using ELISA kits (Aviva System Biology Corp, San Diego, USA) according to the manufacturer’s instructions. Absorbance was quantified at 450 nm by a Multiscan FC Microplate Photometer (Thermo Fisher Scientific).

### Statistical analysis

Statistical tests were performed with the SPSS 26.0 (IBM, Chicago, IL) software. For paired comparisons between groups, the nonparametric, 2-sided Wilcoxon rank-sum test was applied. We applied the nonparametric receiver operating characteristics (ROC) curves to determine the optimal cut-off value with the highest sensitivity and specificity for the prediction of death within 24 months. Survival analyses were done using Kaplan–Meier curves, log-rank test, and univariable Cox proportional hazards regression analysis. For multivariable analysis, Cox regression models were used including parameters with a *p*-value of 0.05 in the univariable analysis. Investigators were blinded to clinical group assignments during the analyses.

## Results

### Identification of proteins with differential expression between Abi sensitive and resistant PCa cells

**Fig. 1:**
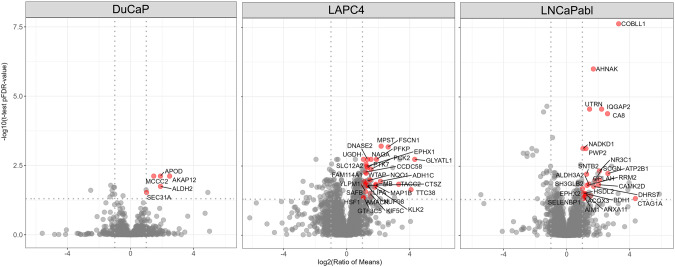
Volcano Plot visualization of differentially abundant proteins comparing the parental (Abi-sensitive) and Abi-resistant cell lines. Red dots indicate the significantly (FDR-corrected *p*-value ≤ 0.05) at least two-fold upregulated proteins in Abi-resistant cells.

We identified 413 (LNCaP vs LNCaP-Abi), 588 (LAPC4 vs LAPC4-Abi) and 172 (DUCAP vs DUCAP-Abi) significantly differentially regulated proteins by at least 2 unique peptides using LC-MS/MS analysis (Supplementary Tables 1–3). Of these above identified proteins, we filtered those which were significantly upregulated in Abi resistant cells and showed at least two-fold higher expression in resistant compared to the parental sensitive PCa cell lines. This step resulted in 25, 38, 5 proteins in LNCaP-Abi, LAPC4-Abi, and DuCaP-Abi cell line pairs. In order to further select the most promising candidate proteins, we used two different bioinformatics workflows. The first, “secreted protein” workflow identified KLK2, while the second “protein scoring” workflow selected FSCN1, CTAG1A and AMACR for further ELISA analyses. FSCN1 reached high score by the “protein scoring” workflow because of its known role in oncological processes. CTAG1A had a higher score as it showed the strongest (25.58-fold) upregulation in LAPC4-Abi-resistant cells. AMARC reached a high score because its molecular interactions (number of edges) according to the STRING database (Fig. 1, Supplementary Fig. 1).

### Selected protein levels in patients’ samples

#### Patients’ characteristics

The patients’ characteristics are given in Table 1.

**Table 1:** Baseline characteristics of Abi and Doc treated patients.

Parameters	Abi	Doc
Total number of patients	100	69
Age (range)	70.8 (52.0–90.0)	70.5 (43.8–86.1)
PSA (ng/mL)	66.5 (0.1–6785.0)	73.7 (3.2–6115.4)
ECOG PS (%)		
0	58 (58)	43 (62)
1	14 (14)	17 (25)
2	0 (0)	9 (13)
unknown	28 (28)	0 (0)
Metastases (%)		
bone	87 (87)	64 (93)
LN (>2 cm)	18 (18)	7 (10)
soft tissue	10 (10)	24 (35)
Primary local therapy (%)		
prostatectomy	43 (43)	14 (20)
radiation	18 (18)	9 (13)
Therapy line (%)		
1st line	44 (44)	62 (90)
2nd line	55 (55)	7 (9)
3rd line	1 (1)	0 (0)
PSA response (%) >30	74 (74)	42 (60)
>50	64 (64)	34 (49)
>90	28 (28)	22 (32)
Number of patients died	69 (69)	55 (80)
OS median, months	19.0	24.1

In the Abi cohort, the median age was 70 (range: 52–90) years, the median pre-treatment PSA value was 66.5 ng/ml. Eighty-seven patients had bone, 17 had lymph node and 10 had visceral metastases. Sixty-nine patients died within a median follow-up period of 19 months.

In the Doc cohort, the median age was 70 years (range: 43–86), the median pre-treatment PSA level was 73 ng/ml. Sixty-four patients had bone, 7 had lymph node and 24 had visceral metastases. Fifty-five of 69 patients died within 24 months.

#### Associations of clinicopathological data with serum FSCN1, KLK2, CTAG1A and AMACR baseline levels

In the Abi cohort, we found no associations between baseline FSCN1 levels and patients’ clinicopathological parameters (Supplementary Table 4). CTAG1A serum levels were significantly lower in patients who showed a 30% or 50% PSA response to Abi (*p* = 0.016, *p* = 0.047, respectively). KLK2 levels were significantly higher in patients who had pain (*p* = 0.012) (Supplementary Table 5). In the Doc cohort, we found significantly lower FSCN1 serum levels in those patients who underwent primary local therapy (radiation or RPE) (*p* = 0.038). Moreover, FSCN1 serum levels were significantly lower in men who had visceral metastases (*p* = 0.027) (Supplementary Table 4). AMACR was undetectable in patients’ serum samples.

#### Survival analyses

**Fig. 2:**
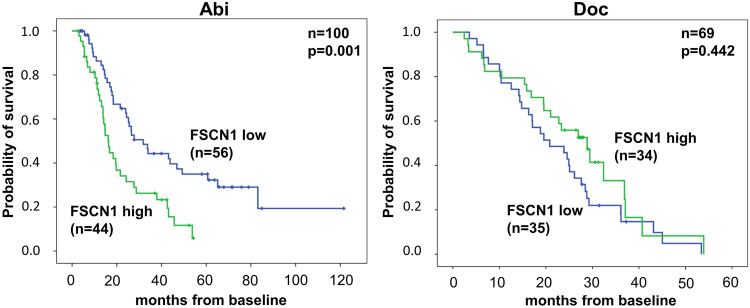
Kaplan–Meier survival curves showed that elevated baseline FSCN1 levels are associated with poor OS in mCRPC men who underwent Abi therapy (left) (*n* = 100). In Doc cohort, FSCN1 showed no significant associations with OS (*n* = 69).

In the Abi cohort, high ECOG status (>1) was associated with shorter overall survival (OS) (*p* < 0.001). High baseline PSA and FSCN1 levels (>9.39 and 10.22 ng/ml by ROC cut-off) were significantly associated with poor OS (*p* < 0.001, *p* = 0.022, *p* = 0.002; respectively) (Table 2). Multivariable analysis revealed that high ECOG status (>1), and high baseline FSCN1 serum levels are independently associated with poor OS in Abi-treated patients (Table 3). Kaplan–Meier OS curve revealed that higher baseline FSCN1 serum levels are significantly associated with poor OS (*p* = 0.001) (Fig. 2). Cancer-specific survival was available only in the Abi cohort. By using this endpoint, high pretreatment FSCN1 serum levels proved to be associated with shorter cancer-specific survival both in the univariable and multivariable analyses (Supplementary Tables 6 and 7) (Supplementary Fig. 2).

**Table 2:** Univariable analysis in patients who underwent Abi or Doc therapy.

		Abi			Doc		
Variables		Overall survival			Overall survival		
		HR	95% CI	*p*	HR	95% CI	*p*
Age	>72 y.	1.577	0.967–2.571	0.068	1.591	0.929–2.723	0.091
ECOG	>1	5.386	2.566–11.351	**<0.001**	1.418	0.824–2.439	0.208
Visceral mets.	pos.	1.031	0.443–2.399	0.944	1.474	0.660–3.293	0.344
LN mets.	pos.	1.344	0.719–2.514	0.354	0.796	0.442–1.433	0.446
Bone mets.	pos.	2.426	0.971–6.058	0.058	2.091	0.506–8.643	0.308
Primary local treatment	pos.	0.600	0.369–0.974	**0.039**	0.996	0.557–1.779	0.988
Primary RPE	pos.	0.804	0.499–1.296	0.370	0.902	0.451–1.804	0.770
Primary RT	pos.	0.729	0.390–1.360	0.321	1.028	0.482–2.189	0.944
PSA median	^a^	2.529	1.535–4.168	**<0.001**	0.866	0.505–1.486	0.601
PSA response	Present	1.351	0.584–3.126	0.483	0.954	0.474–1.918	0.895
PSA response	>30%	0.600	0.341–1.056	0.076	0.993	0.976–1.011	0.460
PSA response	>50%	0.628	0.379–1.042	0.072	0.973	0.946–1.000	0.054
PSA response	>90%	0.597	0.349–1.020	0.059	0.983	0.852–1.134	0.811
FSCN1 median	^a^	1.764	1.086–2.866	**0.022**	0.680	0.394–1.174	0.166
FSCN1 (ROC)	^a^	2.182	1.336–3.564	**0.002**	0.733	0.427–1.258	0.260
CTAG1A (median)	>2.285 pg/ml	0.977	0.912–1.047	0.509	–	–	–
KLK2 (median)	>4.088 ng/ml	1.549	0.963–2.493	0.071	–	–	–

Significant values are indicated in bold.

*LN* Lymph node, *RPE* radical prostatectomy, *RT* radiation therapy.

^a^Abi cohort: median (ng/ml): PSA: 66.45, FSCN1: 9.39 /FSCN1 ROC cut-off: 10.22. Doc cohort: median (ng/ml): PSA: 73.69, FSCN1: 5.84 /FSCN1 ROC cut-off: 5.01.

**Table 3:** Multivariable analysis in patients who underwent Abi treatment.

Overall survival			
	HR	95% CI	*P*
Primary prostate treatment (yes)	0.749	0.400–1.399	0.364
ECOG PS (>1)	5.002	2.257–11.089	**<0.001**
PSA (median) >70.8 ng/ml	1.912	0.980–3.732	0.057
FSCN1 (ROC) >10.22 ng/ml	2.116	1.121–3.994	**0.021**

Significant values are indicated in bold.

In the Doc cohort, we found no associations between the analyzed parameters and patients’ OS, while cancer-specific survival was not available (Table 2, Fig. 2).

#### Prognostic value of FSCN1 level changes during Abi therapy

FSCN1 serum concentrations were measured at 3 months after therapy start and were dichotomized as any increase, at least 20% increase, any decrease and at least 20% decrease. We found no correlations between FSCN1 level changes and OS (Supplementary Table 8).

## Discussion

In the present study, we performed a comparative proteome analysis on three Abi resistant and corresponding parental Abi sensitive PCa cell line pairs. From 68 identified proteins in used Abi resistant cell lines, 4 were selected (FSCN1, KLK2, AMACR, CTAG1A) and further assessed by serum analyses in samples of Abi-treated mCRPC patients. This revealed a significant and independent association between high FSCN1 serum concentrations and poor OS. In contrast, FSCN1 concentrations were not associated with OS in Doc-treated patients. These results suggest that FSCN1 is a potentially predictive serum marker for Abi-treatment in mCRPC.

Our comparative proteome profiling method identified a large number of proteins potentially involved in Abi resistance. Interestingly, when comparing the significant at least two-fold upregulated proteins identified in the three PCa cell line pairs, we found no overlap, which suggests a multifactorial background of Abi resistance. As a consequence, probably rather a larger panel then a single marker will be able to cover all possible resistance mechanisms and so adequately predict Abi resistance.

Using the “secreted protein” selection workflow we selected KLK2 protein. Human KLK2 is a member of the kallikrein protein family, which is involved in several biological processes [16]. Many studies reported that high serum KLK2 levels are associated with high Gleason score and early biochemical recurrence in PCa [17]. In contrast, loss of KLK2 expression in PCa tissue was shown to be associated with the presence of aggressive PCa [16]. Tjon-Kon-Fat et al. analyzed the association of platelet-bound biomarkers for their predictive value in Abi- and in Doc-treated CRPC patients and found high KLK2 levels to be associated with OS and progression-free survival in Abi but not Doc-treated patients [18]. Accordingly, our proteome analysis identified KLK2 as a 3.41-fold upregulated protein in Abi resistant (LAPC4-Abi) cells. In addition, our serum KLK2 analysis in pretreatment samples of Abi-treated patients identified a trend between higher KLK2 serum levels and shorter OS of Abi-treated patients, however this correlation missed to reach the significance level (*p* = 0.071).

The “protein scoring” workflow identified three potential biomarkers. AMACR is an enzyme that is involved in bile acid synthesis and beta-oxidation of branched fatty acids [19]. Previous studies found AMACR protein and mRNA tissue expression to be specific to PCa and therefore suggested AMACR as a highly sensitive diagnostic marker for prostate adenocarcinoma [20, 21]. AMARC may also be associated with Doc resistance of PCa. Yoshizawa et al. showed that combined Doc treatment and AMACR inhibition caused decreased cell proliferation in AR-V7 positive PCa cell line [22]. However, the role of AMACR in Abi resistance is yet to be described. Based on our proteome analysis, AMACR showed a 2.84-fold upregulation in Abi-resistant (LAPC4-Abi) cells, but in our ELISA analyses it was not detectable in patients’ serum samples.

The other protein identified by the “protein scoring” workflow was CTAG1A, which is a cancer testis antigen. The exact biological function of this protein is not well established, although some studies suggest its participation in cell cycle regulation and apoptosis [23]. Grupp et al. analyzed tissue samples of more than 11,000 patients and found high CTAG1A protein expression as an independent predictor of prognosis in ERG-positive PCa [24]. Moreover, it was shown that in ERG-negative cancer, CTAG1A expression is significantly associated with *PTEN* deletion [24]. Based on this, CTAG1A was suggested as a hallmark marker for a separate molecular subgroup of PCa. However, the role of CTAG1A in Abi resistance is unknown. In the present study, we found that CTAG1A is 28.58-fold overexpressed in Abi-resistant (LNCaPabl-Abi) cells, but we did not find a significant correlation between serum CTAG1A concentrations and OS in Abi-treated patients.

The third protein identified by the “protein scoring” workflow was Fascin-1 (FSCN1), which is an actin-binding protein. In vitro study showed that FSCN1 enhanced the migratory capacity of PCa cells [25]. Moreover, an in vivo PCa mouse xenograft study found that inhibition of FSCN1 effectively blocked tumor progression [25]. Based on a systematic review and meta-analysis, high FSCN1 expression is associated with an increased risk of progression in colorectal, breast and esophageal cancer and with the presence of metastatic lesions in gastric and colorectal cancer [26]. In PCa, Darnel et al. analyzed the tissue sample of 196 men who underwent radical prostatectomy and found epithelial FSCN1 expression to be higher in localized and castration resistant PCa compared to benign prostate tissue while no correlation was found between FSCN1 epithelial expression and surgical margins, stage and Gleason score [25]. Furthermore, another study assessed FSCN1 immunostaining in 211 prostate tumors and found that only 8% of the tumors had >10% FSCN1 positive cells. Moreover, they found no significant correlation between FSCN1 expression of tumor cells and pathological stage, Gleason score and PSA levels. However, high stromal FSCN1 expression was significantly associated with high Gleason score [27]. In addition, Tataru et al. compared serum levels of FSCN1 between PCa patients and healthy controls and found no diagnostic value for FSCN1 [28].

FSCN1 may be involved in the development of systemic therapy resistance. FSCN1 plays a crucial role in doxorubicin resistance by facilitating the epithelial-mesenchymal transition (EMT) in hepatocellular carcinoma cells [29]. Similarly, Pan et al. found that FSCN1 participates in EMT and enhances the development of Doc resistance in lung adenocarcinoma cell lines [30]. However, the role of FSCN1 in Abi resistance has not yet been investigated so far. Our comparative proteome analysis revealed a 6,18-fold upregulation of FSCN1 expression in Abi-resistant (LAPC4-Abi) cells. Our ELISA analysis identified baseline FSCN1 serum levels to be independently associated with poor OS of Abi-treated patients. In contrast to Abi-treated patients, we found no correlations between FSCN1 levels and shorter OS in Doc-treated patients, suggesting a therapy specific prognostic value for FSCN1. Based on these results patients with high serum FSCN1 levels may less benefit from Abi than from Doc treatment. Our results however, need further confirmation in larger prospective patient cohorts. Furthermore, the analysis of the potential Abi predictive role of FSCN1 serum levels in metastatic hormone sensitive cases is necessary to assess its value also in this therapeutic setting.

Our study has some limitations inherent from its retrospective nature (e.g. missing data points based on clinical documentation). In addition, only one cohort for Abi and one cohort for Doc treatment was available for analysis, which did not allow us to perform independent validation of our results. Therefore, independent validation preferably in a prospectively collected patient cohort is necessary, before implementing our results in the clinical routine. Moreover, cause of death data was available only for the Abi cohort and none of the Doc cohort and thus cancer-specific survival could only be used as an endpoint in Abi-treated patients. However, if applying cancer-specific survival as an endpoint high serum FSCN1 level was found to be and independent risk-factor. Finally, as we did not perform analyses of already identified resistance markers (e.g. AR-V7) we cannot concurrently evaluate the predictive value of FSCN1. In addition, our in vitro model is rather representative for acquired than for de novo resistance mechanisms, while the assessed serum samples were collected before treatment start (and are therefore rather representative for de novo resistance markers), which is a methodological limitation for this study. However, acquired resistance mechanisms and markers may overlap with those of de novo resistance, therefore our approach is most probably able to identify predictive markers in the pretreatment serum samples.

In conclusion, our results revealed, for the first time, an independent prognostic value of FSCN1 for Abi- but not Doc-treated patients. Based on these, FSCN1 serum level is a promising predictive biomarker for the identification of mCRPC patients with baseline resistance to Abi.

## Supplementary information

Supplementary Materials and Methods

Supplementary Figure 1

Supplementary Figure 2

Supplementary Table 1

Supplementary Table 2

Supplementary Table 3

Supplementary Table 4

Supplementary Table 5

Supplementary Table 6

Supplementary Table 7

Supplementary Table 8

## Data Availability

The raw mass spectrometry data generated in this study have been deposited in the PRIDE database with accession number PXD038697 (http://www.ebi.ac.uk/pride). Other data that support the findings of this study are available from the corresponding author upon request.
