# Correction: Comparative proteome and serum analysis identified FSCN1 as a marker of abiraterone resistance in castration-resistant prostate cancer

**DOI:** 10.1038/s41391-025-01064-6

**Published:** 2025-12-10

**Authors:** Anita Csizmarik, Nikolett Nagy, Dávid Keresztes, Melinda Váradi, Thilo Bracht, Barbara Sitek, Kathrin Witzke, Martin Puhr, Ilona Tornyi, József Lázár, László Takács, Gero Kramer, Sabina Sevcenco, Agnieszka Maj-Hes, Boris Hadaschik, Péter Nyirády, Tibor Szarvas

**Affiliations:** 1 Department of Urology, Semmelweis University, Budapest, Hungary; 2 Department of Molecular Biology, Semmelweis University, Budapest, Hungary; 3 Medizinisches Proteom-Center, Ruhr University Bochum, Bochum, Germany; 4 Department of Anesthesia, Intensive Care Medicine and Pain Therapy, University Hospital Knappschaftskrankenhaus Bochum, Bochum, Germany; 5 Center for Protein Diagnostics, Medical Proteome Analysis, Ruhr-University Bochum, Bochum, Germany; 6 Department of Urology, Medical University of Innsbruck, Innsbruck, Austria; 7 Department of Human Genetics, University of Debrecen, Debrecen, Hungary; 8 Biosystems International Kft, Debrecen, Hungary; 9 Department of Urology, Medical University of Vienna, Vienna, Austria; 10 Department of Urology, University Hospital Essen, University of Duisburg-Essen, Essen, Germany

**Keywords:** Predictive markers, Prostate cancer, Cancer therapy

Correction to: *Prostate Cancer and Prostatic Diseases* 10.1038/s41391-023-00713-y, published online 26 August 2023

In Table 2 of this article, the unit for CTAG1A and KLK2 were incorrect and should have read pg/ml and ng/ml respectively.

The incorrect Table 2

**Table 2:** Univariable analysis in patients who underwent Abi or Doc therapy.

		Abi			Doc		
Variables		Overall survival			Overall survival		
		HR	95% CI	p	HR	95% CI	p
Age	>72 y.	1.577	0.967–2.571	0.068	1.591	0.929–2.723	0.091
ECOG	>1	5.386	2.566–11.351	**<0.001**	1.418	0.824–2.439	0.208
Visceral mets.	pos.	1.031	0.443–2.399	0.944	1.474	0.660–3.293	0.344
LN mets.	pos.	1.344	0.719–2.514	0.354	0.796	0.442–1.433	0.446
Bone mets.	pos.	2.426	0.971–6.058	0.058	2.091	0.506–8.643	0.308
Primary local treatment	pos.	0.600	0.369–0.974	**0.039**	0.996	0.557–1.779	0.988
Primary RPE	pos.	0.804	0.499–1.296	0.370	0.902	0.451–1.804	0.770
Primary RT	pos.	0.729	0.390–1.360	0.321	1.028	0.482–2.189	0.944
PSA median	^a^	2.529	1.535–4.168	**<0.001**	0.866	0.505–1.486	0.601
PSA response	present	1.351	0.584–3.126	0.483	0.954	0.474–1.918	0.895
PSA response	>30%	0.600	0.341–1.056	0.076	0.993	0.976–1.011	0.460
PSA response	>50%	0.628	0.379–1.042	0.072	0.973	0.946–1.000	0.054
PSA response	>90%	0.597	0.349–1.020	0.059	0.983	0.852–1.134	0.811
FSCN1 median	^a^	1.764	1.086–2.866	**0.022**	0.680	0.394–1.174	0.166
FSCN1 (ROC)	^a^	2.182	1.336–3.564	**0.002**	0.733	0.427–1.258	0.260
CTAG1A (median)	>2.285 ng/ml	0.977	0.912–1.047	0.509	-	-	-
KLK2 (median)	>4.088 pg/ml	1.549	0.963–2.493	0.071	-	-	-

Significant values are indicated in bold. LN - Lymph node, RPE – radical prostatectomy, RT – radiation therapy.

^a^Abi cohort: median (ng/ml): PSA: 66.45, FSCN1: 9.39 /FSCN1 ROC cut-off: 10.22. Doc cohort: median (ng/ml): PSA: 73.69, FSCN1: 5.84 /FSCN1 ROC cut-off: 5.01.

The corrected Table 2

**Table 2:** Univariable analysis in patients who underwent Abi or Doc therapy.

		Abi			Doc		
Variables		Overall survival			Overall survival		
		HR	95% CI	p	HR	95% CI	p
Age	>72 y.	1.577	0.967–2.571	0.068	1.591	0.929–2.723	0.091
ECOG	>1	5.386	2.566–11.351	**<0.001**	1.418	0.824–2.439	0.208
Visceral mets.	pos.	1.031	0.443–2.399	0.944	1.474	0.660–3.293	0.344
LN mets.	pos.	1.344	0.719–2.514	0.354	0.796	0.442–1.433	0.446
Bone mets.	pos.	2.426	0.971–6.058	0.058	2.091	0.506–8.643	0.308
Primary local treatment	pos.	0.600	0.369–0.974	**0.039**	0.996	0.557–1.779	0.988
Primary RPE	pos.	0.804	0.499–1.296	0.370	0.902	0.451–1.804	0.770
Primary RT	pos.	0.729	0.390–1.360	0.321	1.028	0.482–2.189	0.944
PSA median	^a^	2.529	1.535–4.168	**<0.001**	0.866	0.505–1.486	0.601
PSA response	present	1.351	0.584–3.126	0.483	0.954	0.474–1.918	0.895
PSA response	>30%	0.600	0.341–1.056	0.076	0.993	0.976–1.011	0.460
PSA response	>50%	0.628	0.379–1.042	0.072	0.973	0.946–1.000	0.054
PSA response	>90%	0.597	0.349–1.020	0.059	0.983	0.852–1.134	0.811
FSCN1 median	^a^	1.764	1.086–2.866	**0.022**	0.680	0.394–1.174	0.166
FSCN1 (ROC)	^a^	2.182	1.336–3.564	**0.002**	0.733	0.427–1.258	0.260
CTAG1A (median)	>2.285 pg/ml	0.977	0.912–1.047	0.509	-	-	-
KLK2 (median)	>4.088 ng/ml	1.549	0.963–2.493	0.071	-	-	-

Significant values are indicated in bold. LN - Lymph node, RPE – radical prostatectomy, RT – radiation therapy.

^a^Abi cohort: median (ng/ml): PSA: 66.45, FSCN1: 9.39 /FSCN1 ROC cut-off: 10.22 Doc cohort: median (ng/ml): PSA: 73.69, FSCN1: 5.84 /FSCN1 ROC cut-off: 5.01.

The original article has been corrected.

