# Supplementary material for: Comparative proteome and serum analysis identified FSCN1 as a marker of abiraterone resistance in castration-resistant prostate cancer
Source: Prostate Cancer Prostatic Dis. 2023 Aug 26;27(3):451–6. doi: 10.1038/s41391-023-00713-y (PMC11319194; doi:10.1038/s41391-023-00713-y)
Supplement: Supplementary file 1 — Supplementary Materials and Methods [file 41391_2023_713_MOESM1_ESM.docx]

**Supplementary Materials and Methods**

**Detailed description of LC-MS/MS analysis**

**Sample preparation of cells for Label-free LC-MS/MS**

Abi sensitive (parental) and resistant PC cell lines were harvested at about 80% confluence. The cell pellets were lysated using 0.1% NaDOC in TBS supplemented with protease inhibitors (complete mini, Roche, Penzberg, Germany) and Benzonase (25 U per sample, Merck). Then, the samples were sonicated on ice for 10 min and lysis buffer was added for protein solubilization (30 mM Tris, 7M urea, 2 M thiourea, 0.1% SDS, pH 8.5). The samples were centrifugated at 16100 x g for 10 min. The supernatant concentrations were measured using the Bradford assay (Bio-Rad, Hercules, CA). 30 µg of proteins were loaded to 18% Tris-Glycine-Gels (Anamed Elektrophorese, Rodau, Germany) and allowed to run into the gel (15 min at 100 V). The protein bands were stained with Coomassie and cut from the gels. In-gel trypsin digestion was performed in 10 mM ammonium bicarbonate buffer overnight at 37 °C. The generated peptides were extracted in a vacuum centrifuge and peptides were dissolved in 0.1% TFA. We measured peptide concentrations via amino acid analysis as described before (Megger et al., Mol Cell Proteomics. 2013 Jul;12(7):2006-20.).

**LC-MS/MS Parameters**

LC–MS/MS analysis was performed as described previously (Megger et al., Mol Cell Proteomics. 2013 Jul;12(7):2006-20.). Briefly, 300 ng tryptic digested proteins were injected to an Ultimate 3000 RSLCnano HPLC coupled to an Orbitrap Elite instrument (both Thermo Sientific, Bremen, Germany). Peptides were concentrated on a C18 trap column (Acclaim PepMap 100; 100 μm × 2 cm, 5 μm, 100 Å) at a flow rate of 30 μl/min with 0.1% TFA for 7 min. Peptides were transferred to a Nano Viper C18 analytical column (Acclaim PepMap RSLC; 75 μm × 50 cm, 2 μm, 100 Å) and separated with a gradient from 5%–40% solvent B over 98 min at 400 nl/min and 60°C (solvent A: 0.1% FA; solvent B: 0.1% FA, 84% ACN). Full-scan mass spectra were operated in profile mode at a resolution of 60,000 at 400 m/z within a mass range of 350–2000 m/z. MS/MS spectra were acquired at a resolution of 5,400. For MS/MS measurements, the 20 most abundant peptide ions were fragmented by collision-induced dissociation (CID, NCE 35).

## **Protein Identification and Quantification**

For protein identification, we used the Proteome Discoverer v.1.4 (Thermo Fisher Scientific). Spectra were searched against the UniProtKB/Swiss-Prot database (Release 2016_05; 70625 entries) using Mascot v.2.5 (Matrix Science, London, UK). The following search parameters were applied: Homo sapiens taxonomy, precursor ion mass tolerance of 5 ppm and fragment ion mass tolerance of 0.4 Da, dynamic and static modifications methionine (oxidation) and cysteine (carbamidomethyl). The false discovery rate (FDR) was estimated with the Target Decoy PSM Validator and identifications with an FDR > 1% were rejected. We used Progenesis QI v.2.0.5387.52102 (Nonlinear Dynamics, Durham, NC, USA) for label-free quantification. Raw files were aligned to a reference run and a master map of features was applied to all experimental runs to adjust for differences in retention time. Ion charge states of 2+, 3+, and 4+ with a minimum of three isotope peaks were considered. Statistical analysis was done using R and t-tests were calculated using arcsinh-transformed normalized protein abundances. We determined the ratios of mean abundances (RoM) based on non-transformed data. Proteins quantify with minimum two unique peptides and those passing the applied significance thresholds (p-value < 0.05) were considered as significantly differentially abundant.
