# Supplementary material for: Comparative proteome and serum analysis identified FSCN1 as a marker of abiraterone resistance in castration-resistant prostate cancer
Source: Prostate Cancer Prostatic Dis. 2023 Aug 26;27(3):451–6. doi: 10.1038/s41391-023-00713-y (PMC11319194; doi:10.1038/s41391-023-00713-y)
Supplement: Supplementary file 7 — Supplementary Table 4 [file 41391_2023_713_MOESM7_ESM.docx]

**Supplementary Table 4**. Association of pretreatment FSCN1 levels with clinicopathological parameters in Abi and Doc-treated patients. Significant values are indicated in bold. RPE – radical prostatectomy, RT – radiation therapy, PS –Performance Status. Significant values are indicated in bold.

|  |  | **Abi** |  |  | **Doc** |  |
| --- | --- | --- | --- | --- | --- | --- |
|  | n | FSCN1 serum cc. (ng/ml) | P | n | FSCN1 serum cc. (ng/ml) | P |
|  |  | median (range) |  |  | median (range) |  |
| whole cohort | 100 | 9.39 (0.00 - 24.44) |  | 69 | 5.84 (1.39 - 21.30) |  |
|  |  |  |  |  |  |  |
| Age (years) |  |  |  |  |  |  |
| ≤ 72 | 57 | 8.69 (0.00 - 24.44) | 0.231 | 39 | 5.81 (1.39 - 14.32) | 0.753 |
| > 72 | 43 | 10.00 (1.92 - 22.32) |  | 30 | 5.93 (1.58 - 21.30) |  |
| Primary therapy |  |  |  |  |  |  |
| no | 40 | 10.22 (0.00 - 24.44) | 0.699 | 48 | 6.16 (1.38 - 21.30) | **0.038** |
| yes | 60 | 9.18 (1.01 - 23.53) |  | 21 | 4.07 (1.58 - 11.31) |  |
| Primary RPE |  |  |  |  |  |  |
| no | 57 | 10.39 (0.00 - 24.44) | 0.142 | 55 | 5.84 (1.39 - 21.30) | 0.332 |
| yes | 43 | 7.76 (1.01 - 22.32) |  | 14 | 4.90 (1.58 - 11.31) |  |
| Primary RT |  |  |  |  |  |  |
| no | 82 | 8.75 (0.00 - 24.44) | 0.075 | 60 | 6.02 (1.39 - 21.30) | 0.487 |
| yes | 18 | 10.87 (4.68 - 23.53) |  | 9 | 5.07 (2.20 - 11.31) |  |
| ECOG PS |  |  |  |  |  |  |
| 0 | 58 | 9.19 (0.00 - 24.44) | 0.255 | 43 | 5.84 (1.39 - 21.30) | 0.901 |
| 1-2 | 14 | 5.84 (1.01 - 20.32) |  | 26 | 5.94 (1.58 - 13.93) |  |
| unknown | 28 |  |  | 0 |  |  |
| Lymph node status |  |  |  |  |  |  |
| N - | 82 | 9.38 (0.73 - 24.44) | 0.507 | 45 | 5.79 (1.58 - 21.30) | 0.791 |
| N + | 18 | 10.47 (0.00 - 23.53) |  | 24 | 6.06 (1.39 - 12.73) |  |
| unknown |  |  |  | 0 |  |  |
| Visceral mets. |  |  |  |  |  |  |
| no | 89 | 9.38 (0.00 - 24.44) | 0.844 | 62 | 6.02 (1.58 - 21.30) | **0.027** |
| yes | 10 | 8.24 (2.70 - 20.32) |  | 7 | 3.19 (1.39 - 13.93) |  |
| unknown | 1 |  |  | 0 |  |  |
| Bone mets. |  |  |  |  |  |  |
| no | 13 | 9.47 (0.00 - 18.80) | 0.766 | 5 | 6.09 (1.58 - 10.67) | 0.779 |
| yes | 87 | 9.38 (0.73 - 24.44) |  | 64 | 5.83 (1.39 - 21.30) |  |
| unknown |  |  |  | 0 |  |  |
| PSA response |  |  |  |  |  |  |
| response | 89 | 9.43 (0.00 - 24.44) | 0.943 | 52 | 5.91 (1.39 - 21.30) | 0.394 |
| no response | 11 | 8.99 (3.96 - 18.79) |  | 13 | 6.10 (2.50 - 12.73) |  |
| unknown |  |  |  | 4 |  |  |
| PSA response |  |  |  |  |  |  |
| > 30% | 74 | 9.35 (0.00 - 24.44) | 0.360 | 42 | 5.80 (1.39 - 21.30) | 0.158 |
| < 30% | 26 | 9.37 (3.15 - 23.53) |  | 23 | 6.10 (2.50 - 17.00) |  |
| unknown | 0 |  |  | 4 |  |  |
| PSA response |  |  |  |  |  |  |
| > 50% | 64 | 9.13 (0.00 - 24.00) | 0.396 | 34 | 5.14 (1.39 - 21.30) | 0.156 |
| < 50% | 36 | 9.37 (1.92 - 23.53) |  | 31 | 6.60 (1.58 - 17.00) |  |
| unknown | 0 |  |  | 4 |  |  |
| PSA response |  |  |  |  |  |  |
| > 90% | 28 | 8.79 (1.01 - 20.32) | 0.417 | 22 | 5.14 (1.39 - 21.30) | 0.239 |
| < 90% | 72 | 9.47 (0.00 - 24.44) |  | 43 | 6.10 (1.58 - 17.00) |  |
| unknown | 0 |  |  | 4 |  |  |
