# Supplementary material for: Comparative proteome and serum analysis identified FSCN1 as a marker of abiraterone resistance in castration-resistant prostate cancer
Source: Prostate Cancer Prostatic Dis. 2023 Aug 26;27(3):451–6. doi: 10.1038/s41391-023-00713-y (PMC11319194; doi:10.1038/s41391-023-00713-y)
Supplement: Supplementary file 8 — Supplementary Table 5 [file 41391_2023_713_MOESM8_ESM.docx]

**Supplemetary Table 5** Association of baseline CTAG1A and KLK2 levels with clinicopathological parameters in Abi-treated patients. Significant values are indicated in bold. RPE – radical prostatectomy, RT – radiation

|  | n | CTAG1A serum cc. (pg/ml) | P | KLK2 serum cc. (ng/ml) | P |
| --- | --- | --- | --- | --- | --- |
|  |  | median (range) |  | median (range) |  |
| whole cohort | 100 | 2.24 (0.10 - 10.58) |  | 4.09 (0.30 - 17.83) |  |
|  |  |  |  |  |  |
| Age (years) |  |  |  |  |  |
| ≤ 72 | 57 | 2.23 (0.10 - 10.58) | 0.873 | 3.81 (0.30 - 17.83) | 0.587 |
| > 72 | 43 | 2.29 (0.28 - 7.28) |  | 4.53 (0.40 - 17.83) |  |
| Primary therapy |  |  |  |  |  |
| no | 40 | 2.24 (0.24 - 5.93) | 0.852 | 6.09 (0.30 - 17.83) | 0.257 |
| yes | 60 | 2.23 (0.10 - 10.58) |  | 3.43 (0.40 - 17.83) |  |
| Primary RPE |  |  |  |  |  |
| no | 57 | 2.29 (0.19 - 5.93) | 0.854 | 4.53 (0.30 - 17.83) | 0.865 |
| yes | 43 | 2.01 (0.10 - 10.58) |  | 3.45 (0.56 - 17.83) |  |
| Primary RT |  |  |  |  |  |
| no | 82 | 2.22 (0.10 - 10.58) | 0.750 | 4.45 (0.30 - 17-83) | 0.360 |
| yes | 18 | 2.64 (0.19 - 5.48) |  | 3.61 (0.40 - 11.87) |  |
| ECOG PS |  |  |  |  |  |
| 0 | 58 | 2.22 (0.10 - 10.58) | 0.989 | 3.35 (0.30 - 17.83) | 0.147 |
| 1-2 | 14 | 2.07 (0.32 - 5.97) |  | 5.76 (1.15 - 17.83) |  |
| unknown | 28 |  |  |  |  |
| Pain |  |  |  |  |  |
| no | 47 | 2.01 (0.10 - 6.40) | 0.106 | 2.86 (0.40 - 17.83) | ***0.012*** |
| yes | 33 | 2.53 (0.57 - 10.58) |  | 5.96 (1.01 - 17.83) |  |
| unknown | 20 |  |  |  |  |
| Lymph node status |  |  |  |  |  |
| N - | 82 | 2.20 (0.10 - 10.58) | 0.435 | 4.09 (0.30 - 17.83) | 0.858 |
| N + | 18 | 2.30 (0.57 - 5.48) |  | 4.17 (0.93 - 14.77) |  |
| Visceral mets. |  |  |  |  |  |
| no | 89 | 2.26 (0.10 - 10.58) | 0.501 | 4.52 (0.30 - 17.83) | 0.454 |
| yes | 10 | 1.82 (0.47 - 5.97) |  | 2.25 (0.73 - 17.83) |  |
| unknown | 1 |  |  |  |  |
| Bone mets. |  |  |  |  |  |
| no | 13 | 2.30 (0.47 - 5.22) | 0.810 | 2.81 (0.93 - 12.85) | 0.495 |
| yes | 87 | 2.17 (0.10 - 10.58) |  | 4.37 (0.30 - 17.83) |  |
| PSA response |  |  |  |  |  |
| response | 89 | 2.23 (0.10 - 10.58) | 0.800 | 4.36 (0.30 - 17.83) | 0.437 |
| no response | 11 | 2.50 (0.71 - 3.91) |  | 3.47 (0.40 - 8.73) |  |
| PSA response |  |  |  |  |  |
| > 30% | 74 | 2.04 (0.10 - 10.58) | ***0.016*** | 3.43 (0.30 - 17.83) | 0.295 |
| < 30% | 26 | 3.26 (0.71 - 7.81) |  | 4.53 (1.20 - 17.83) |  |
| PSA response |  |  |  |  |  |
| > 50% | 64 | 2.19 (0.10 - 10.58) | ***0.047*** | 4.09 (0.56 - 17.83) | 0.912 |
| < 50% | 36 | 2.58 (0.65 - 7.81) |  | 3.81 (0.30 - 17.83) |  |
| PSA response |  |  |  |  |  |
| > 90% | 28 | 1.40 (0.28 - 10.58) | 0.091 | 2.69 (0.56 - 17.83) | 0.267 |
| < 90% | 72 | 2.26 (0.10 - 7.81) |  | 4.36 (0.30- 17.83) |  |
