# Supplementary material for: Comparative proteome and serum analysis identified FSCN1 as a marker of abiraterone resistance in castration-resistant prostate cancer
Source: Prostate Cancer Prostatic Dis. 2023 Aug 26;27(3):451–6. doi: 10.1038/s41391-023-00713-y (PMC11319194; doi:10.1038/s41391-023-00713-y)
Supplement: Supplementary file 9 — Supplementary Table 6 [file 41391_2023_713_MOESM9_ESM.docx]

**Supplementary Table 6:** Univariable analysis of CCS in patients who underwent Abi therapy. Significant values are indicated in bold. LN - Lymph node, RPE – radical prostatectomy, RT – radiation therapy

|  |  | **Abi** | | | |
| --- | --- | --- | --- | --- | --- |
| Variables |  | Overall survival | | | |
|  |  | HR | 95% CI | | p |
| Age | >72 y. | 1.653 | 0.974 - 2.745 | 0.063 | |
| ECOG | >1 | 5.055 | 2.262 - 11.294 | | **>0.001** |
| Visceral mets. | pos. | 1.168 | 0.499 - 2.736 | | 0.721 |
| LN mets. | pos. | 1.640 | 0.830 - 3.241 | | 0.155 |
| Bone mets. | pos. | 2.142 | 0.853 - 5.380 | | 0.105 |
| Primary local treatment | pos. | 0.592 | 0.354 - 0.991 | | **0.046** |
| Primary RPE | pos. | 0.781 | 0.470 - 1.300 | | 0.342 |
| Primary RT | pos. | 0.757 | 0.393 - 1.456 | | 0.404 |
| PSA median | 66.45 ng/ml | 2.704 | 1.584 - 4.616 | | **<0.001** |
| PSA response | present | 1.179 | 0.506 - 2.743 | | 0.703 |
| PSA response | >30% | 0.549 | 0.304 - 0.990 | | **0.046** |
| PSA response | >50% | 0.635 | 0.370 - 1.089 | | 0.099 |
| PSA response | >90% | 0.609 | 0.345 - 1.076 | | 0.088 |
| FSCN1 median | 9.39 ng/ml | 1.774 | 1.060 - 2.971 | | **0.029** |
| FSCN1 (ROC) | 10.22 ng/ml | 2.138 | 1.272 - 3.594 | | **0.004** |
| CTAG1A (median) | > 2.285 ng/ml | 1.139 | 0.688 - 1.886 | | 0.613 |
| KLK2 (median) | > 4.088 pg/ml | 1.492 | 0.901 - 2.473 | | 0.120 |
