# Supplementary material for: Comparative proteome and serum analysis identified FSCN1 as a marker of abiraterone resistance in castration-resistant prostate cancer
Source: Prostate Cancer Prostatic Dis. 2023 Aug 26;27(3):451–6. doi: 10.1038/s41391-023-00713-y (PMC11319194; doi:10.1038/s41391-023-00713-y)
Supplement: Supplementary file 10 — Supplementary Table 7 [file 41391_2023_713_MOESM10_ESM.docx]

**Supplementary Table 7**: Multivariable analysis of CSS in patients who underwent Abi treatment. Significant values are indicated in bold.

|  |  | **Overall survival** |  |
| --- | --- | --- | --- |
|  | HR | 95% CI | P |
| Primary prostate treatment (yes) | 0.786 | 0.407 - 1.520 | 0.475 |
| ECOG PS (>1) | 4.249 | 1.829 - 9.872 | **0.001** |
| PSA (median) >70.8 ng/ml | 2.548 | 1.243 - 5.220 | **0.011** |
| FSCN1 (ROC) > 10.22 ng/ml | 1.944 | 0.999 - 3.783 | **0.050** |
