# Supplementary material for: Comparative proteome and serum analysis identified FSCN1 as a marker of abiraterone resistance in castration-resistant prostate cancer
Source: Prostate Cancer Prostatic Dis. 2023 Aug 26;27(3):451–6. doi: 10.1038/s41391-023-00713-y (PMC11319194; doi:10.1038/s41391-023-00713-y)
Supplement: Supplementary file 11 — Supplementary Table 8 [file 41391_2023_713_MOESM11_ESM.docx]

**Supplementary Table 8:** Univariable OS analysis in Abi cohort based on FSCN1 marker changes in the whole cohort

|  |  |  | **Overall survival** | | | |
| --- | --- | --- | --- | --- | --- | --- |
|  | **increase** | **n** | HR | 95% CI | | P |
| 3 months | no | 23 | ref. |  |  |  |
|  | any | 17 | 1.041 | 0.521 - 2.081 | | 0.909 |
|  | < 20% | 7 | ref. |  |  |  |
|  | > 20% | 10 | 1.633 | 0.812 - 3.284 | | 0.169 |
|  | **decrease** | **n** |  |  | |  |
| 3 months | no | 17 | ref. |  |  |  |
|  | any | 23 | 0.921 | 0.457 - 1.858 | | 0.819 |
|  | < 20% | 7 | ref. |  |  |  |
|  | > 20% | 16 | 0.890 | 0.384 - 2.064 | | 0.786 |
